# Leveraging 13 million responses to the U.S. COVID-19 Trends and Impact Survey to examine vaccine hesitancy, vaccination, and mask wearing, January 2021-February 2022

**DOI:** 10.1186/s12889-022-14286-3

**Published:** 2022-10-13

**Authors:** Quynh C. Nguyen, Isha Yardi, Francia Ximena Marin Gutierrez, Heran Mane, Xiaohe Yue

**Affiliations:** 1 Department of Epidemiology and Biostatistics, University of Maryland School of Public Health, 4254 Stadium Dr. , 20742 College Park, MD USA; 2 Department of Behavioral and Community Health, University of Maryland School of Public Health, College Park, MD USA

**Keywords:** COVID-19 vaccine hesitancy, COVID-19 vaccine, Mask wearing behavior, Health surveys, Big data, Social media

## Abstract

**Background:**

The urgency of the COVID-19 pandemic called upon the joint efforts from the scientific and private sectors to work together to track vaccine acceptance and prevention behaviors.

**Methods:**

Our study utilized individual responses to the Delphi Group at Carnegie Mellon University U.S. COVID-19 Trends and Impact Survey, in partnership with Facebook. We retrieved survey data from January 2021 to February 2022 (n = 13,426,245) to examine contextual and individual-level predictors of COVID-19 vaccine hesitancy, vaccination, and mask wearing in the United States. Adjusted logistic regression models were developed to examine individual and ZIP code predictors of COVID-19 vaccine hesitancy and vaccination status. Given the COVID-19 vaccine was rolled out in phases in the U.S. we conducted analyses stratified by time, January 2021-May 2021 (Time 1) and June 2021-February 2022 (Time 2).

**Results:**

In January 2021 only 9% of U.S. Facebook respondents reported receiving the COVID-19 vaccine, and 45% were vaccine hesitant. By February 2022, 80% of U.S. Facebook respondents were vaccinated and only 18% were vaccine hesitant. Individuals who were older, held higher educational degrees, worked in white collar jobs, wore a mask most or all the time, and identified as white and Asian had higher COVID-19 vaccination rates and lower vaccine hesitancy across Time 1 and Time 2. Essential workers and blue-collar occupations had lower COVID vaccinations and higher vaccine hesitancy. By Time 2, all adults were eligible for the COVID-19 vaccine, but blacks and multiracial individuals had lower vaccination and higher vaccine hesitancy compared to whites. Those 55 years and older and females had higher odds of wearing masks most or all the time. Protective service, construction, and installation and repair occupations had lower odds of wearing masks. ZIP Code level percentage of the population with a bachelors’ which was associated with mask wearing, higher vaccination, and lower vaccine hesitancy.

**Conclusion:**

Associations found in earlier phases of the pandemic were generally found to also be present later in the pandemic, indicating stability in inequities. Additionally, inequities in these important outcomes suggests more work is needed to bridge gaps to ensure that the burden of COVID-19 risk does not disproportionately fall upon subgroups of the population.

## Background

On January 20th, 2020, the Centers for Disease Prevention and Control (CDC) confirmed the first case of the COVID-19 virus in the United States [1]. As of April 21, 2022, COVID-19 has globally taken over 6.2 million lives and infected over 504 million individuals [2]. The urgency of the COVID-19 global pandemic called upon the scientific community to implement public health policy measures and expedite the development and distribution of a universal vaccine.

The CDC released its first mask recommendation on April 3, 2020 to curb the spread of COVID-19 [3]. Across the duration of the pandemic, 39 states and Puerto Rico and Washington DC required people to wear masks in public. However, 11 states did not have any mask mandates at any point and some states such as Florida, Iowa, Montana, Tennessee and Texas utilized legislation or executive action to prevent local governments from implementing mask mandates. Approximately 32 states lifted indoor mask mandates after the pandemic eased in the summer of 2021 [4]. As of May 2022, no states are broadly required mask wearing in public, although some states mandate mask wearing in high risk settings such as healthcare and long-term care facilities [4]. Remaining mask mandates and policies varied by city and demographic requirements, such as some areas extending their mask mandates specifically for individuals below a certain age [4]. In places with no state or local mask requirements, businesses and private establishments were able to institute their own mask policies.

Shortly after masking policies were implemented, the first vaccines against COVID-19 were made available to a select few individuals. On December 11th, 2020, the Food and Drug Administration (FDA) approved the use of Pfizer-BioNTech COVID-19 vaccine under emergency use authorization for individuals 16 years and older [5]. Soon thereafter, the Moderna and Johnson & Johnson COVID-19 vaccines were approved for emergency use [6]. COVID-19 vaccines help prevent infections, symptomatic illness, hospitalization, and death [7]. They also work to protect against COVID-19 variants and while vaccinated individuals may experience breakthrough infections, COVID-19 vaccines help prevent severe illness and mortality [8]. According to the CDC, as of April 2022, a total of 567 million vaccine doses were administered in the US. About 77% of the U.S population received at least one dose and 66% were fully vaccinated, and 99 million booster doses had been administered which constituted only 50% of the total booster-eligible population [9].

Despite the scientific data on efficacy and safety, scientists and public health professionals quickly recognized that COVID-19 vaccine hesitancy would present major challenges in combating the pandemic [10–13]. Vaccine hesitancy has been a palpable roadblock to getting individuals vaccinated in the United States. The US Household Pulse Survey that found almost 50% of vaccine hesitant individuals were concerned about potential vaccine side effects and 40% of vaccine hesitant individuals simply did not trust the COVID-19 vaccine or harbored skepticism towards the vaccine’s efficacy [14]. The more than two year-long global pandemic has likely exacerbated traditional reasons for hesitancy observed with other vaccines [15]. The COVID-19 pandemic has largely been characterized by hostile political undertones and the spreading of misinformation which can lead to more hesitancy towards getting the vaccine. Public surveys identified political affiliation [16, 17] and government trust [18] as influencing vaccine hesitancy, with individuals citing those who endorse the vaccine [19], geographical origin of the vaccine [20], and political motives playing a role in their attitudes towards the COVID-19 vaccine. Regarding COVID-19 vaccination mandates, only 22 states have instituted a vaccine mandate, with a majority of these states being concentrated on the east and west coasts of the country [21]. States included in this number have vaccination mandates listed either for all individuals or for certain demographics such as school employees and healthcare workers. 15 states do not have any vaccine mandate in place, while 14 states prohibit the passage of any COVID-19 vaccine mandates [21, 22].

The combination of vaccination and mask wearing can work synergistically protect against COVID-19, since immunity from vaccines wanes over time [23]. Nonetheless, although studies [23] suggest that the strongest defense against COVID-19 is the combination of full vaccination and masking wearing, data indicates that some individuals are hesitant about adopting one or both and that these behaviors might be correlated [24, 25]. A previous study found that people with positive vaccine intentions were more likely to wear masks [24]. Therefore, examining both masking and vaccine hesitancy in tandem can help to contextualize hesitancy regarding multiple COVID-19 prevention strategies.

### Study aims and study hypotheses

The goal of this project is to understand COVID-19 related behaviors including COVID-19 vaccination, vaccine hesitancy, and mask wearing in the United States and explore individual-level and contextual characteristics that significantly predict these beliefs and behaviors. This study utilized over 13 million individual responses to the U.S. COVID-19 Trends and Impact Survey collected from January 2021 to February 2022. We hypothesized that older individuals, females, higher education groups, and white collar occupations will have lower COVID-19 vaccine hesitancy and higher COVID-19 vaccination rates and mask wearing. At the ZIP code level, we hypothesized that communities with higher socioeconomic status and greater urban development will have lower vaccine hesitancy and higher COVID-19 vaccination rates and mask wearing. Additionally, given that the COVID vaccine was rolled out in phases in the United States with only certain population groups gaining access to the vaccine in stages, we examined patterns in COVID-19 vaccination before and after it was available to the general population. We hope that these results can be used to inform the development of policies and programs to help protect all individuals from coronavirus.

## Methods

In collaboration with Facebook, along with a consortium of universities and public health officials, the Delphi group at Carnegie Mellon University conducted the U.S. COVID-19 Trends and Impact Survey (CTIS) to monitor the spread and impact of the COVID-19 pandemic in the United States. The survey was advertised through Facebook. It ran daily from April 6, 2020, to June 25, 2022, and about 40,000 people in the United States participated each day. The sampling frame was Facebook users aged 18 years or older who had been active on the platform in the last month. Participants were recruited for the survey through an advertisement placed in their Facebook news feed. Facebook automatically selected a random sample of its users daily to see the advertisements. Users who clicked on the ad were taken to the online Qualtrics survey that was administered by Carnegie Mellon University. Facebook did not receive their survey responses. The survey was available in various languages including English, Spanish, Brazilian Portuguese, Vietnamese, French, and Chinese. To account for differences between Facebook users and the United States population, Carnegie Mellon provided Facebook with study identifiers of people who completed the survey. Facebook then calculated survey weights indicating how representative each person is of the United States population based upon demographic data known to Facebook. These survey weights can then be applied in analyses of the data to produce nationally representative results. Importantly throughout the survey process, confidentiality of individuals’ data was protected. Carnegie Mellon cannot use the participant IDs to identify specific Facebook users and Facebook never received the individual survey responses [26, 27].

Survey participants reported on COVID-19 symptoms, COVID-19 testing, vaccination status, vaccine hesitancy, mask wearing, health behaviors, demographic and family characteristics. We obtained the data through a restricted data access agreement with Carnegie Mellon that enabled us to have individual-level response data with ZIP code identifiers and used individual survey responses from January 2021-February 2022 (n = 13,426,245). Our study was approved by the Institutional Review Board at the University of Maryland College Park. Below, we provided details on survey questions utilized.

### COVID-19 vaccination, vaccine hesitancy, and mask wearing

COVID-19 vaccination status was assessed by asking, “Have you had a COVID-19 vaccination?“ Those who responded with “yes” were coded as having received a COVID-19 vaccine. The survey also asked respondents the number of doses they have received, but given the survey was implemented daily on different cross-sections of the United States population and our study period was from January 2021 to February 2022, not all individuals would have the opportunity to receive two or more doses. Thus, our analyses examined whether individuals received at least one dose of a COVID-19 vaccine.

COVID-19 Vaccine hesitancy was only assessed among participants who did not have at least one dose of a COVID-vaccine. For these participants who indicated they had not yet received the COVID-19 vaccine, they were subsequently asked “If a vaccine to prevent COVID-19 were offered to you today, would you choose to get vaccinated?” Participants were given four response options: (1) “Yes, definitely,” (2) “Yes, probably,” (3) “No, probably not,” (4) “No, definitely not.” In our analyses, participants selecting options 2–4 were coded as “vaccine hesitant” while those who responded with option 1 were categorized as “not vaccine hesitant.”

If the participant answered with anything other than “Yes, definitely” they would choose to get vaccinated, a question appeared asking them to select from the following vaccine hesitancy reasons: I am concerned about possible side effects of a COVID-19 vaccine; I don’t know if a COVID-19 vaccine will work; I don’t believe I need a COVID-19 vaccine; I don’t like vaccines; I plan to wait and see if it is safe and may get it later; I think other people need it more than I do right now; I am concerned about the cost of a COVID-19 vaccine; I don’t trust the government; It is against my religious beliefs; Other. Among those who are vaccine hesitant, we examined the top reasons reported for why respondents did not receive the COVID-19 vaccine.

Mask wearing in public. Respondents were asked “In the past 5 days, how often did you wear a mask when in public?” Responses included “All the time”, “Most of the time”, “Some of the time”, “A little of the time”, “None of the time”, and “I have not been in public during the past 5 days”. We examined predictors of wearing masks most or all the time.

### Individual level covariates

Individual-level characteristics included in regression analyses included COVID-19 symptoms (“In the past 24 hours, have you or anyone in your household had any of the following symptoms, fever, sore throat, cough, shortness of breath, difficulty breathing), Age (categories into the following groups 18–24 years, 25–34 years, 35–44 years, 45–54 years, 55–64 years, 65–74 years, 75 years or older), Race/ethnicity (White, Hispanic, Black, Asian, American Indian/Alaska Native, Native Hawaiian/Pacific Islander, Multiple race, Unknown race), Travel outside state (“In the past 7 days, have you traveled outside of your state?”), Occupation type (Community and social service; Education, library services; Arts, entertainment, media; Healthcare practitioners; Healthcare support; Protective service; Food preparation and serving; Building/grounds cleaning & maintenance; Personal care & service; Sales; Office & admin support; Construction; Installation & repair; Production; Transportation & material moving; Other occupation; Unemployed in past 4 weeks), User Language (English, Other), Highest Education Degree (Less than high school, High school graduate or equivalent(GED), Some college, 2 year degree, 4 year degree, master’s degree, Professional degree, Doctorate), Gender (male, female, others), and Family size (number of people in household).

### ZIP code level variables

Analyses also examined the association between neighborhood characteristics (operationalized at the ZIP code level) and COVID-19 health behaviors. ZIP code level variables were obtained from the American Community Survey (ACS) 2018 5-year estimates and included median age, median household income, percentage black, percent Hispanic, percentage with a bachelor’s degree, and civilian employment rate. ZIP code built environment characteristics were created utilizing computer vision on Google Street View images. Images were processed using trained Visual Geometry Group (VGG-19 model) deep convolutional networks (previously detailed by Nguyen et al. [28–30]) to identify the built environment features of interest which included presence of sidewalk and mixed land use (mixture of buildings other than detached single family homes) with accuracies of 85% for sidewalks and 82% for mixed land use.

## Analytic approach

We estimated the prevalence of vaccine hesitancy among survey respondents and top vaccine hesitancy reasons. We graphed temporal trends in vaccine hesitancy, vaccination status, and mask wearing. Adjusted logistic regression models were developed to examine predictors of COVID-19 vaccine hesitancy, vaccination status and mask wearing, controlling for individual level and ZIP code level potential confounders. COVID-19 symptoms were included as a control variable. Regression analyses were run separately for two time periods; January 2021-May 2021 (Time 1) and June 2021-February 2022 (time 2). Time 1 was characterized by greater limitations in COVID-19 vaccine eligibility and vaccine supplies. Time 2 saw individuals 5 years and older qualify for the COVID-19 vaccine and more availability in COVID-19 vaccination. All survey analyses were weighted to correct for sampling bias and produce nationally representative estimates. Stata MP15 (StataCorp LP, College Station, TX) was used for all statistical analyses. This study was approved by the University of Maryland Institutional Review Board.

## Results

Table 1 presents descriptive statistics of survey respondents from January 2020 to February 2022. Respondents came from a variety of age groups with seemingly adequate representation from the younger and older groups. For example, 26% of respondents were 18–35 years old and 22% were 65 years and older (Table 1). About 40% had a bachelor’s degree or higher. 52% were female, 44% were male and 4% reported other gender. About 7% reported a user language other than English. About 4% worked in the food industry, 5% in education, and 7% in healthcare. About 43% reported not having worked for pay in the past 4 weeks.

**Table 1:** Descriptive Statistics of survey respondents from the U.S. COVID-19 Trends and Impact Survey, Jan 2021-Feb 2022

*Respondent characteristics*	N	% (95% CI)/ Mean (SD)
COVID-19 vaccine hesitant	12,944,975	22.90 (22.86, 22.93)
COVID-19 vaccinated (at least 1 dose)	13,058,980	67.33 (67.29, 67.36)
Age 18–24	11,679,657	10.26 (10.22, 10.29)
Age 25–34	11,679,657	15.78 (15.75, 15.81)
Age 35–44	11,679,657	16.58 (16.55, 16.60)
Age 45–54	11,679,657	17.18 (17.16, 17.21)
Age 55–64	11,679,657	17.95 (17.93, 17.98)
Age 65–74	11,679,657	15.17 (15.14, 15.19)
Age 75 or older	11,679,657	7.09 (7.07, 7.10)
< High school	11,506,734	4.39 (4.37, 4.40)
High school	11,506,734	18.79 (18.76, 18.82)
Some college	11,506,734	25.30 (25.26, 25.33)
2 year degree	11,506,734	11.05 (11.03, 11.07)
4 year degree	11,506,734	22.83 (22.80, 22.86)
Master’s degree	11,506,734	3.12 (3.11, 3.14)
Professional degree	11,506,734	2.46 (2.45, 2.47)
Doctorate	11,506,734	12.06 (12.04, 12.08)
Has COVID-like symptoms	13,824,925	21.08 (21.05, 21.10)
Male	11,721,409	44.38 (44.34, 44.42)
Female	11,721,409	51.29 (51.25, 51.33)
Other gender	11,721,409	4.33 (4.31, 4.35)
White	13,843,328	54.08 (54.05, 54.12)
Hispanic	13,843,328	13.81 (13.79, 13.84)
Black	13,843,328	5.40 (5.38, 5.41)
Asian	13,843,328	2.39 (2.37, 2.40)
American Indian/Alaska Native	13,843,328	0.74 (0.74, 0.75)
Native Hawaiian/Pacific Islander	13,843,328	0.20 (0.20, 0.21)
Multiple race	13,843,328	4.44 (4.43, 4.46)
Unknown race	13,843,328	18.93 (18.90, 18.96)
Other language vs. English	13,843,328	7.27 (7.25, 7.29)
Community and social service	11,193,962	2.23 (2.22, 2.24)
Education, library occupation	11,193,962	4.73 (4.71, 4.74)
Arts, entertainment, media	11,193,962	1.82 (1.81, 1.83)
Healthcare practitioners	11,193,962	4.50 (4.49, 4.52)
Healthcare support	11,193,962	2.98 (2.97, 2.99)
Protective service	11,193,962	0.88 (0.87, 0.89)
Food preparation and serving	11,193,962	3.77 (3.75, 3.79)
Building/grounds cleaning & maintenance	11,193,962	1.34 (1.33, 1.35)
Personal care & service	11,193,962	1.14 (1.13, 1.14)
Sales	11,193,962	4.99 (4.97, 5.01)
Office & admin support	11,193,962	6.06 (6.05, 6.08)
Construction	11,193,962	1.45 (1.44, 1.46)
Installation & repair	11,193,962	2.02 (2.01, 2.04)
Production	11,193,962	1.65 (1.64, 1.66)
Transportation & material moving	11,193,962	2.60 (2.58, 2.61)
Other occupation	11,193,962	15.03 (15.01, 15.06)
Unemployed in past 4 weeks	11,193,962	42.81 (42.77, 42.85)
Travelled outside the state	11,703,670	13.17 (33.81)
Wear mask all the time	12,305,558	48.98 (48.95, 49.02)
Wear mask most of the time	12,305,558	17.02 (16.99, 17.04)
Wear mask some of the time	12,305,558	9.61 (9.59, 9.63)
Wear mask a little of the time	12,305,558	7.32 (7.30, 7.34)
Wear mask none of the time	12,305,558	14.16 (14.13, 14.18)
Have not been in public	12,305,558	2.92 (2.91, 2.93)
Family size	13,426,245	3.59 (5.54)
*ZIP code characteristics*		
Population density	12,808,117	3329 (8374)
Median age	12,808,117	50.83 (2.70)
Median income	12,808,117	64,178 (25,449)
% of population holding a bachelor’s degree	12,808,117	0.31 (0.16)
Employment rate for civilians	12,808,117	0.94 (0.03)
% Black	12,808,117	0.11 (0.16)
% Hispanic	12,808,117	0.17 (0.20)
% Asian	12,808,117	0.05 (0.08)
Sidewalks	12,808,117	0.39 (0.27)
Mixed land use	12,808,117	0.27 (0.20)

Descriptive statistics were estimated using sampling weights to produce nationally representative estimates. Weighted means and standard errors were calculated for continuous variables. Weighted proportions and Wald 95% confidence intervals were calculated for proportions.

**Fig. 1:**
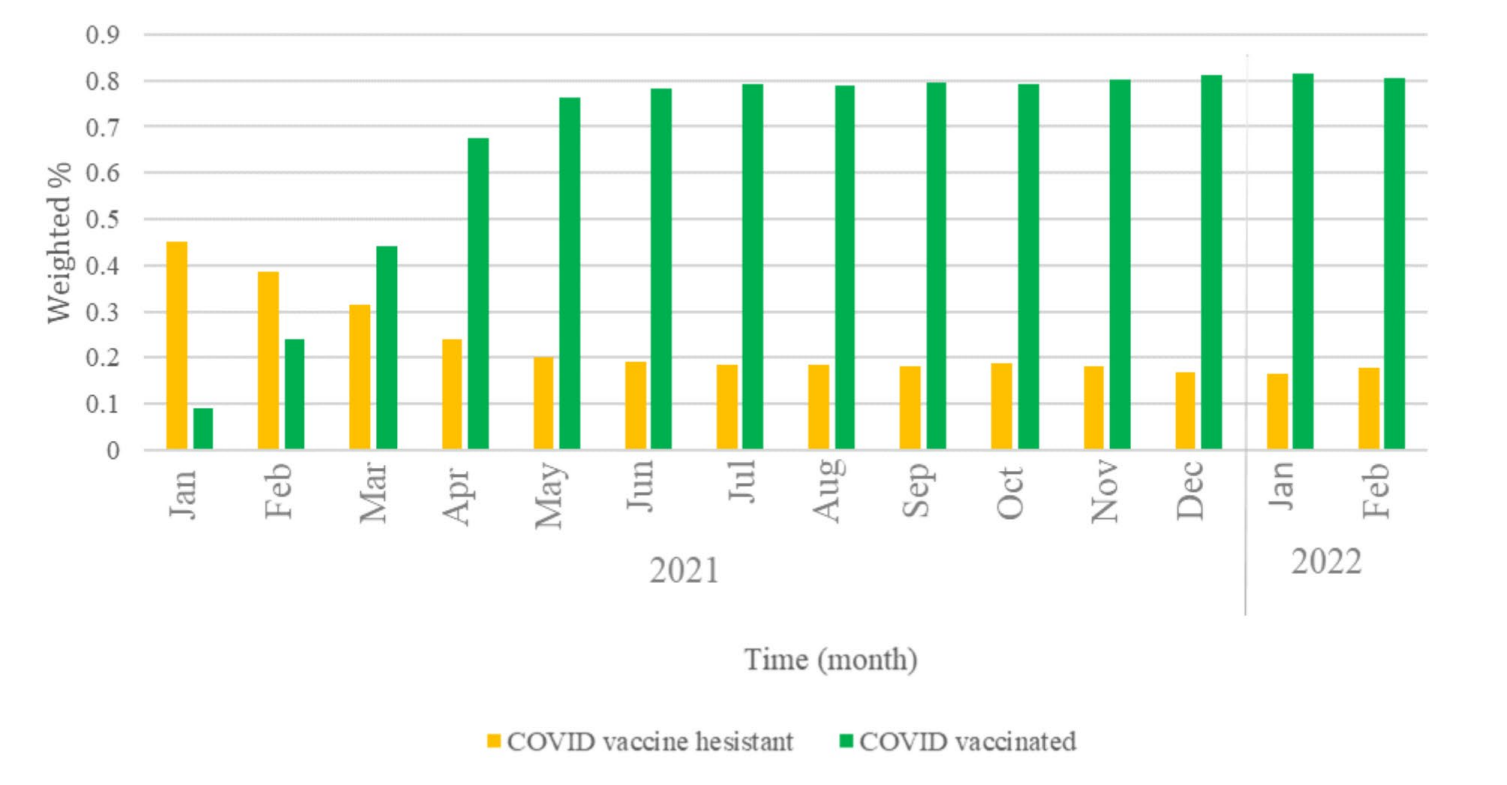
**COVID-19 vaccination and COVID-19 vaccine hesitancy by time, January 2021-Febrary 2022.** COVID vaccinated were U.S. Facebook survey respondents who reported having gotten at least one dose of a COVID-19 vaccine. COVID-19 vaccine hesitant were individuals who indicated that they would “No, probably not” and “No, definitely not” get the COVID vaccine if it was offered to them today. X-axis indicates study month from January 2021 and to February 2022

Figure 1 displays temporal trends in COVID-19 vaccinations and COVID-19 vaccine hesitancy. In January 2021 (month 1 of our study), about 9% of U.S. Facebook respondents had received at least one dose of a COVID-19 vaccine. COVID-19 vaccinations increased quickly to 44% by March 2021 and 68% by April 2021. By February 2022 (month 14), COVID-19 vaccinations had reached 80% among U.S. Facebook respondents. Across the study time period, we saw COVID-19 vaccine hesitancy decrease. On January 2021, 45% reported being COVID-19 vaccine hesitant. By March 2021, vaccine hesitancy had decreased to 31% and was 24% in April 2021. It continued to decrease and by Feb 2022 it was 18% (Fig. 1).

**Fig. 2:**
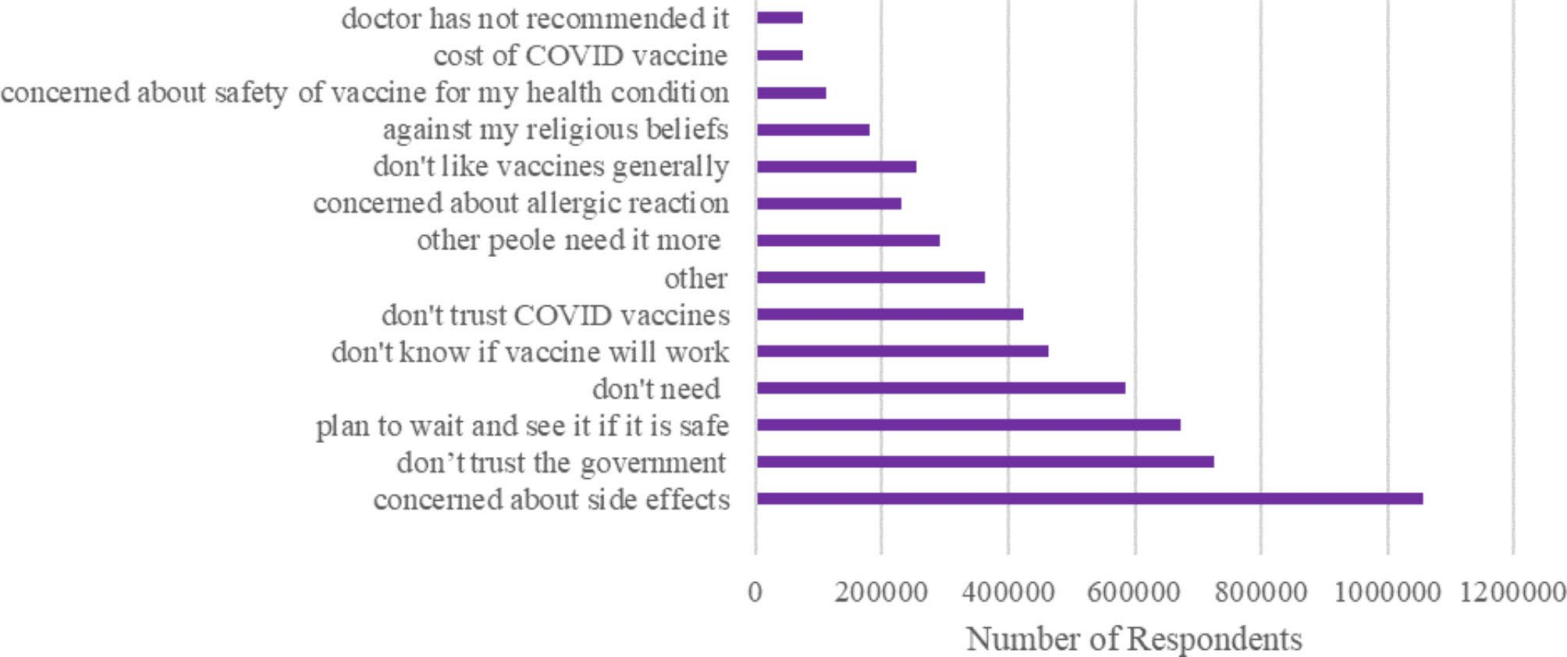
**COVID-19 Vaccine Hesitancy Reason, Jan 2021-Feb 2022.** Hesitancy reasons among vaccine hesitant individuals. Respondents could select multiple reasons

Among the vaccine hesitant, the top five reasons for being hesitant about getting the COVID-19 vaccine include the following: concerned about vaccine side effects (19%), not trusting the government (13%), planned to wait and see whether the vaccine is safe (12%), believed they did not need the vaccine (11%), and don’t know if the vaccine will work (8%) (Fig. 2).

**Fig. 3:**
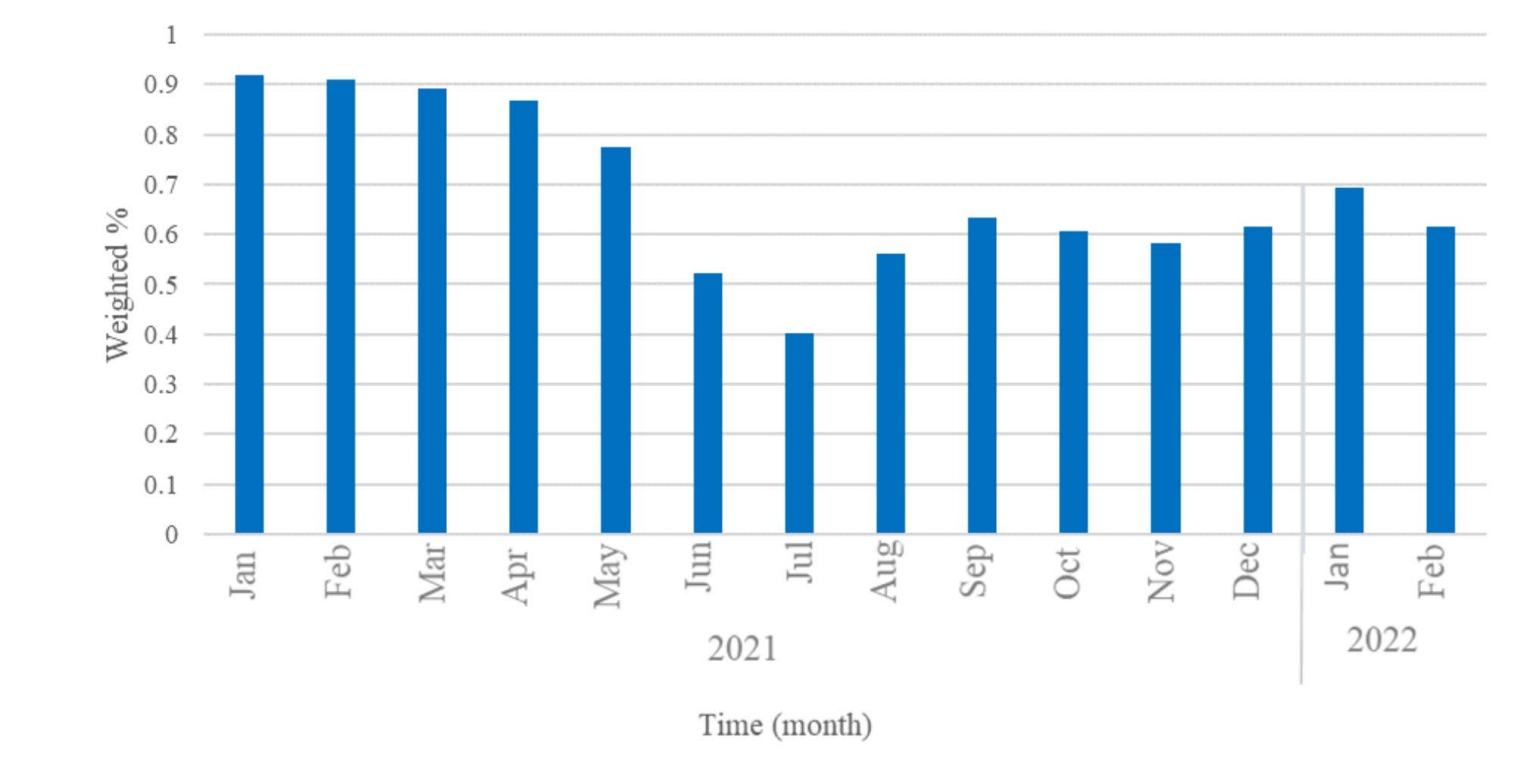
**Mask wearing most/all the time in public (Jan 2021-Feb 2022).** Temporal trends in the prevalence of U.S. Facebook survey respondents reporting they were masks most or all of the time in public. X-axis indicates study month from January 2021 and to February 2022

Masking-wearing fluctuated across the time period. In January 2021, 91% reported wearing masks most or all the time in public. Mask wearing steadily decreased as COVID-19 vaccination increased and was 40% in July 2021. As COVID-19 variants emerged, mask wearing increased and was 62% in February 2022 (Fig. 3).

Table 2 displays adjusted logistic regression model results. In big data, statistical significance is much more likely when the data size grows very large, and hence statistical significance might not be as useful in interpreting results of big datasets. Hence in our description of results, we point to the magnitude and precision of estimated associations. Our results suggest that older people were more likely to be vaccinated: for example, respondents who were 65 years or older were 3–5 times more likely to be fully vaccinated compared to those aged 18–24 years old. The data also suggests that older people were less likely to show COVID-19 vaccine hesitancy: respondents who were 75 years or older had an OR as low as 0.21 (95% CI: 0.21, 0.22) in Time 1 and OR = 0.24 ((95% CI: 0.24, 0.25) in Time 2, which means that their odds of vaccine hesitancy were 76–79% lower compared to those aged 18–24 years old. Respondents who had a bachelors’ degree or higher were more likely to be vaccinated than lower education groups and they were about 60% less likely to COVID-19 vaccine hesitant. Respondents who worked in the healthcare industry were more likely to be vaccinated and showed less vaccine hesitancy in Time (1) However, this relationship disappeared in Time (2) At Time 1, COVID-19 vaccinations were lower among essential workers and blue-collar occupations (ORs ranged from 0.31 to 0.40), including those in food preparation and serving, construction, installation and repair, transportation, and production (Table 2). In Time 2, these disparities attenuated (moved closer to the null value) but were still present (OR = 0.36 to 0.64). For these same occupation groups, vaccine hesitancy was higher (OR = 1.88–2.30 in Time 1) and (OR = 2.05–2.80 in Time 2) (Table 2).

Across the two time periods, lower frequency of mask wearing in public was associated with increasing vaccine hesitancy and lower vaccination in a graded fashion. Reporting wearing masks only ‘some of the time’, ‘little of the time’, and ‘none the time’ was associated with increasing odds of vaccine hesitancy and lower vaccination compared to those who reported wearing masks most or all the time. For instance, wearing masks a little of the time (OR = 0.51–0.55) or none of the time (OR = 0.19–0.28) were associated with lower COVID-19 vaccination. Wearing masks a little of the time or none of the time was associated with 6–12 times higher odds of vaccine hesitancy in Time 1 and 2–6 times higher odds in Time 2 (Table 2). The majority of ZIP code level contextual characteristics did not strongly predict COVID-19 vaccination status or vaccine hesitancy. The strongest predictor was ZIP code level percentage of the population with a bachelors’ which was associated with higher vaccination and lower vaccine hesitancy.

**Table 2:** Time stratified models for COVID-19 vaccination and vaccine hesitancy

	At least one dose of COVID vaccine		Vaccine hesitancy	
	Jan to May 2021	June to February 2022	Jan to May 2021	June to February 2022
*Respondent characteristics*	OR (95% CI)	OR (95% CI)	OR (95% CI)	OR (95% CI)
Age 18–24	1.00	1.00	1.00	1.00
Age 25–34	1.12 (1.10, 1.14)	0.93 (0.91, 0.94)	1.04 (1.03, 1.06)	1.12 (1.10, 1.14)
Age 35–44	1.35 (1.33, 1.37)	1.16 (1.14, 1.18)	0.84 (0.83, 0.85)	0.90 (0.89, 0.92)
Age 45–54	1.48 (1.46, 1.51)	1.42 (1.39, 1.44)	0.71 (0.70, 0.72)	0.74 (0.72, 0.75)
Age 55–64	1.88 (1.86, 1.91)	2.06 (2.03, 2.10)	0.49 (0.48, 0.50)	0.50 (0.49, 0.51)
Age 65–74	3.66 (3.60, 3.71)	3.39 (3.33, 3.46)	0.28 (0.28, 0.28)	0.30 (0.29, 0.31)
Age 75 or older	5.10 (5.02, 5.19)	3.98 (3.89, 4.06)	0.21 (0.21, 0.22)	0.24 (0.24, 0.25)
< High school	1.00	1.00	1.00	1.00
High school	1.18 (1.16, 1.20)	1.23 (1.21, 1.26)	0.92 (0.91, 0.94)	0.85 (0.83, 0.87)
Some college	1.45 (1.42, 1.47)	1.61 (1.58, 1.64)	0.69 (0.68, 0.70)	0.67 (0.65, 0.68)
2 year degree	1.53 (1.50, 1.56)	1.62 (1.58, 1.65)	0.66 (0.64, 0.67)	0.67 (0.66, 0.69)
4 year degree	1.87 (1.84, 1.91)	2.53 (2.48, 2.59)	0.41 (0.40, 0.42)	0.43 (0.42, 0.44)
Master’s degree	2.05 (2.01, 2.09)	2.08 (2.02, 2.14)	0.36 (0.35, 0.36)	0.52 (0.51, 0.54)
Professional degree	1.84 (1.79, 1.88)	1.53 (1.48, 1.57)	0.38 (0.37, 0.39)	0.70 (0.68, 0.72)
Doctorate	2.06 (2.02, 2.09)	2.80 (2.74, 2.86)	0.33 (0.33, 0.34)	0.39 (0.38, 0.40)
Has COVID-like symptoms	0.59 (0.58, 0.59)	0.81 (0.81, 0.82)	1.41 (1.40, 1.42)	1.16 (1.15, 1.17)
Male	1.00	1.00	1.00	1.00
Female	1.03 (1.03, 1.04)	1.00 (1.00, 1.01)	1.27 (1.26, 1.28)	1.01 (1.01, 1.02)
Other gender	0.83 (0.81, 0.85)	0.53 (0.53, 0.54)	1.42 (1.39, 1.45)	1.88 (1.84, 1.91)
White	1.00	1.00	1.00	1.00
Hispanic	0.98 (0.97, 0.99)	0.93 (0.91, 0.94)	1.16 (1.15, 1.17)	1.03 (1.01, 1.04)
Black	0.86 (0.85, 0.87)	0.71 (0.70, 0.72)	1.82 (1.80, 1.84)	1.30 (1.28, 1.32)
Asian	1.31 (1.29, 1.34)	2.34 (2.24, 2.45)	0.83 (0.81, 0.85)	0.39 (0.37, 0.40)
American Indian/Alaska Native	1.09 (1.06, 1.12)	0.71 (0.69, 0.74)	1.21 (1.18, 1.25)	1.35 (1.31, 1.40)
Native Hawaiian/Pacific Islander	0.89 (0.84, 0.94)	0.69 (0.65, 0.74)	1.42 (1.34, 1.51)	1.34 (1.25, 1.45)
Multiple race	0.82 (0.81, 0.83)	0.47 (0.47, 0.48)	1.74 (1.71, 1.76)	2.13 (2.10, 2.16)
Unknown race	0.88 (0.86, 0.90)	0.54 (0.53, 0.55)	1.54 (1.50, 1.58)	1.77 (1.72, 1.81)
Other language vs. English	0.99 (0.97, 1.00)	1.48 (1.45, 1.51)	0.81 (0.80, 0.82)	0.57 (0.55, 0.58)
Community and social service	1.00	1.00	1.00	1.00
Education, library occupation	0.82 (0.81, 0.84)	1.30 (1.26, 1.34)	0.91 (0.89, 0.93)	0.78 (0.75, 0.80)
Arts, entertainment, media	0.53 (0.51, 0.54)	1.05 (1.01, 1.09)	0.97 (0.94, 1.00)	0.95 (0.91, 0.98)
Healthcare practitioners	1.89 (1.86, 1.93)	1.03 (1.00, 1.06)	0.72 (0.70, 0.74)	0.99 (0.96, 1.02)
Healthcare support	1.35 (1.32, 1.38)	1.05 (1.02, 1.09)	0.83 (0.81, 0.86)	0.95 (0.91, 0.98)
Protective service	0.74 (0.71, 0.76)	0.58 (0.55, 0.60)	1.46 (1.40, 1.52)	1.76 (1.69, 1.84)
Food preparation and serving	0.57 (0.55, 0.58)	0.75 (0.72, 0.77)	1.37 (1.33, 1.41)	1.31 (1.27, 1.35)
Building/grounds cleaning & maintenance	0.49 (0.48, 0.51)	0.56 (0.54, 0.58)	1.59 (1.54, 1.65)	1.77 (1.71, 1.85)
Personal care & service	0.53 (0.52, 0.55)	0.65 (0.62, 0.67)	1.48 (1.44, 1.53)	1.55 (1.49, 1.61)
Sales	0.40 (0.39, 0.40)	0.63 (0.61, 0.65)	1.74 (1.70, 1.78)	1.58 (1.54, 1.63)
Office & admin support	0.56 (0.55, 0.57)	1.01 (0.98, 1.04)	1.18 (1.16, 1.21)	0.99 (0.97, 1.02)
Construction	0.31 (0.30, 0.33)	0.36 (0.34, 0.37)	2.30 (2.22, 2.38)	2.80 (2.70, 2.90)
Installation & repair	0.34 (0.34, 0.35)	0.39 (0.37, 0.40)	2.29 (2.22, 2.37)	2.61 (2.52, 2.70)
Production	0.36 (0.35, 0.37)	0.64 (0.61, 0.66)	1.96 (1.90, 2.02)	1.56 (1.51, 1.62)
Transportation & material moving	0.40 (0.39, 0.41)	0.49 (0.48, 0.51)	1.88 (1.83, 1.94)	2.05 (1.98, 2.11)
Other occupation	0.48 (0.47, 0.49)	0.67 (0.65, 0.68)	1.43 (1.40, 1.47)	1.51 (1.47, 1.55)
Unemployed in past 4 weeks	0.48 (0.47, 0.49)	0.67 (0.66, 0.69)	1.51 (1.48, 1.54)	1.42 (1.39, 1.46)
Travelled outside the state	1.20 (1.19, 1.21)	0.84 (0.83, 0.85)	1.02 (1.01, 1.04)	1.22 (1.21, 1.24)
Wear mask all the time	1.00	1.00	1.00	1.00
Wear mask most of the time	1.30 (1.29, 1.31)	1.12 (1.11, 1.13)	1.55 (1.53, 1.56)	0.93 (0.92, 0.95)
Wear mask some of the time	1.04 (1.02, 1.05)	0.80 (0.79, 0.81)	2.99 (2.95, 3.03)	1.37 (1.35, 1.39)
Wear mask a little of the time	0.55 (0.54, 0.56)	0.51 (0.51, 0.52)	6.26 (6.15, 6.37)	2.21 (2.18, 2.24)
Wear mask none of the time	0.28 (0.27, 0.28)	0.19 (0.19, 0.19)	12.11 (11.88, 12.34)	5.97 (5.91, 6.04)
Have not been in public	0.39 (0.39, 0.40)	0.35 (0.35, 0.36)	1.94 (1.92, 1.97)	2.89 (2.83, 2.94)
Family size	0.99 (0.99, 0.99)	0.98 (0.98, 0.98)	1.01 (1.01, 1.01)	1.02 (1.02, 1.02)
*ZIP code characteristics*				
Population density	0.99 (0.99, 1.00)	1.02 (1.01, 1.02)	1.01 (1.01, 1.01)	0.98 (0.98, 0.98)
Median age	0.99 (0.98, 1.00)	0.96 (0.95, 0.97)	1.03 (1.02, 1.04)	1.04 (1.03, 1.05)
Median income	0.97 (0.97, 0.97)	1.03 (1.02, 1.03)	1.03 (1.03, 1.04)	0.98 (0.98, 0.99)
% of population holding a bachelor’s degree	1.12 (1.11, 1.12)	1.37 (1.36, 1.38)	0.75 (0.75, 0.75)	0.72 (0.72, 0.73)
Employment rate for civilians	1.02 (1.02, 1.03)	1.01 (1.01, 1.02)	0.98 (0.97, 0.99)	0.98 (0.97, 0.99)
% Black	1.01 (1.00, 1.01)	0.99 (0.99, 0.99)	1.00 (1.00, 1.01)	1.01 (1.00, 1.01)
% Hispanic	1.04 (1.04, 1.04)	1.04 (1.03, 1.04)	0.95 (0.95, 0.96)	0.96 (0.96, 0.97)
% Asian	1.01 (1.01, 1.01)	1.01 (1.00, 1.01)	0.99 (0.99, 0.99)	0.99 (0.99, 1.00)
Sidewalks	1.01 (1.00, 1.01)	1.14 (1.13, 1.14)	0.94 (0.94, 0.94)	0.88 (0.88, 0.88)
Presence of apartments/ commercial buildings	1.01 (1.00, 1.01)	0.97 (0.96, 0.97)	0.98 (0.98, 0.99)	1.03 (1.03, 1.04)
N	4,444,201	5,209,755	4,438,960	5,185,128

Individual-level data came from the U.S. COVID-19 Trends and Impact Survey, Jan 2021-Feb 2022. N = number of individual survey responses included in the models. ZIP code demographics data came from American Community Survey (ACS) 2018 5-year estimates and ZIP code built environment data came from neighborhood characteristics derived from Google Street View images. Adjusted logistic regression models for vaccination status and vaccine hesitancy were run separately and stratified by time period. All models controlled simultaneously for all listed covariates. Odds ratios and robust standard errors were calculated to examine associates between predictors and outcomes. Sampling weights were used to produce nationally representative estimates.

Table 3 shows time stratified models results of predictors of mask wearing in public ‘most’ or ‘all the time.’ Study results suggest that older people are more likely to wear masks most or all the time across both time periods compared to young adults (Age 18–24 as reference group). For respondents who showed COVID-like symptoms, they were more likely to wear masks most or all the time, as expected, across two time periods. Females had higher odds of wearing masks most or all the time compared to males across the two time periods. In general, all other race groups were more likely to wear masks most or all the time compared to white respondents, and among them, black respondents showed over three times the odds. For different occupation types, compared to community and social service, protective service (OR = 0.44; 95% CI: 0.42, 0.46 in Time 1), construction (OR = 0.30; 95% CI: 0.29, 0.31 in Time 1) and installation and repair (OR = 0.39; 95% CI: 0.37, 0.40 in Time 1) had much lower odds of wearing masks most or all the time. Most examined ZIP code characteristics did have statistically significant associations with mask wearing. Percent Hispanic and sidewalks were associated with higher odds of wearing masks most or all the time. However, civilian employment rate and mixed land use was associated with lower odds of mask wearing.

**Table 3:** Time stratified models of predictors of mask wearing in public

	Wear mask “most” or “all the time”	
	Jan to May 2021	June to February 2022
*Respondent characteristics*	OR (95% CI)	OR (95% CI)
Age 18–24	1.00	1.00
Age 25–34	0.94 (0.92, 0.96)	0.99 (0.98, 1.01)
Age 35–44	1.02 (0.99, 1.04)	1.12 (1.10, 1.14)
Age 45–54	1.04 (1.02, 1.06)	1.11 (1.10, 1.13)
Age 55–64	1.26 (1.24, 1.29)	1.32 (1.30, 1.34)
Age 65–74	1.41 (1.38, 1.45)	1.42 (1.40, 1.44)
Age 75 or older	1.26 (1.23, 1.29)	1.27 (1.25, 1.29)
< High school	1.00	1.00
High school	1.03 (1.00, 1.06)	0.84 (0.82, 0.85)
Some college	1.08 (1.05, 1.11)	0.83 (0.82, 0.85)
2 year degree	1.00 (0.97, 1.03)	0.80 (0.79, 0.82)
4 year degree	1.17 (1.14, 1.20)	0.82 (0.80, 0.83)
Master’s degree	0.97 (0.93, 1.00)	0.84 (0.83, 0.86)
Professional degree	0.70 (0.68, 0.73)	0.82 (0.80, 0.84)
Doctorate	1.20 (1.16, 1.23)	0.87 (0.85, 0.89)
Has COVID-like symptoms	1.17 (1.15, 1.18)	1.30 (1.29, 1.31)
Male	1.00	1.00
Female	2.06 (2.04, 2.08)	1.77 (1.76, 1.77)
Other gender	0.49 (0.48, 0.50)	1.02 (1.01, 1.04)
White	1.00	1.00
Hispanic	1.60 (1.57, 1.63)	1.59 (1.58, 1.61)
Black	3.34 (3.23, 3.44)	3.51 (3.45, 3.56)
Asian	2.88 (2.73, 3.03)	2.65 (2.59, 2.71)
American Indian/Alaska Native	1.19 (1.14, 1.24)	1.54 (1.50, 1.58)
Native Hawaiian/Pacific Islander	1.60 (1.42, 1.80)	2.23 (2.10, 2.37)
Multiple race	0.64 (0.63, 0.65)	0.97 (0.96, 0.98)
Unknown race	0.68 (0.66, 0.70)	1.10 (1.08, 1.12)
Other language vs. English	3.06 (2.92, 3.19)	2.21 (2.17, 2.25)
Community and social service	1.00	1.00
Education, library occupation	1.34 (1.29, 1.39)	1.09 (1.07, 1.11)
Arts, entertainment, media	1.03 (0.98, 1.08)	1.03 (1.01, 1.06)
Healthcare practitioners	1.06 (1.03, 1.10)	1.08 (1.06, 1.10)
Healthcare support	1.18 (1.14, 1.23)	1.11 (1.08, 1.13)
Protective service	0.44 (0.42, 0.46)	0.49 (0.47, 0.50)
Food preparation and serving	1.03 (0.99, 1.08)	0.88 (0.86, 0.90)
Building/grounds cleaning & maintenance	0.63 (0.60, 0.66)	0.69 (0.67, 0.71)
Personal care & service	0.76 (0.73, 0.80)	0.81 (0.79, 0.83)
Sales	0.70 (0.67, 0.72)	0.70 (0.68, 0.71)
Office & admin support	0.89 (0.86, 0.92)	0.78 (0.77, 0.80)
Construction	0.30 (0.29, 0.31)	0.35 (0.34, 0.36)
Installation & repair	0.39 (0.37, 0.40)	0.47 (0.46, 0.48)
Production	0.68 (0.66, 0.71)	0.64 (0.63, 0.66)
Transportation & material moving	0.59 (0.56, 0.61)	0.64 (0.63, 0.66)
Other occupation	0.69 (0.67, 0.72)	0.74 (0.72, 0.75)
Unemployed in past 4 weeks	1.06 (1.03, 1.10)	1.07 (1.06, 1.09)
Travelled outside the state	0.38 (0.37, 0.38)	0.58 (0.58, 0.58)
Family size	0.98 (0.98, 0.98)	0.99 (0.99, 0.99)
*ZIP code characteristics*		
Population density	1.03 (1.03, 1.04)	1.03 (1.02, 1.03)
Median age	1.04 (1.03, 1.05)	1.05 (1.04, 1.06)
Median income	1.04 (1.04, 1.05)	1.01 (1.01, 1.02)
% of population holding a BA	1.26 (1.25, 1.27)	1.18 (1.18, 1.19)
Employment rate for civilians	0.81 (0.80, 0.82)	0.76 (0.76, 0.77)
% Black	1.03 (1.03, 1.04)	1.14 (1.14, 1.15)
% Hispanic	1.16 (1.15, 1.16)	1.27 (1.26, 1.27)
% Asian	1.05 (1.05, 1.06)	1.15 (1.15, 1.16)
Sidewalks	1.29 (1.28, 1.30)	1.10 (1.10, 1.11)
Mixed land use	0.85 (0.84, 0.85)	0.92 (0.92, 0.92)
N	4,282,805	5,067,621

Individual-level data came from the U.S. COVID-19 Trends and Impact Survey, Jan 2021-Feb 2022. N = number of individual survey responses included in the models. ZIP code demographics data came from American Community Survey (ACS) 2018 5-year estimates and ZIP code built environment data came from neighborhood characteristics derived from Google Street View images. Adjusted logistic regression models for mask wearing were stratified by time period. All models controlled simultaneously for all listed covariates. Odds ratios (OR) and 95% confidence intervals (CI) were calculated to examine associates between predictors and mask wearing. Sampling weights were used to produce nationally representative estimates.

## Discussion

Study findings indicate that age was a major predictor for COVID-19 vaccine uptake, with higher odds of vaccine uptake increasing with age. Older age groups are likely to have more comorbidities, underlying health conditions, and physiological changes that accompany the aging process. The increased risk of severe illness from COVID-19 may incentivize older adults to take the COVID-19 vaccine as a preventative health measure, resulting in higher COVID-19 vaccine uptake and lower vaccine hesitancy [31]. Additionally, older adults were among the first groups eligible to receive the COVID-19 and thus early access may have assisted with vaccine uptake. According to a report by the CDC, individuals between the ages of 5 and 11 had the lowest rates of vaccinations, with 33.6% having received at least one dose and 26.6% having been fully vaccinated [32]. On the other hand, individuals older than 65 years presented the highest rates of vaccination. 95.0% of individuals between the ages of 65 and 74 have received at least one dose, 91.2% have been fully vaccinated and 65.2% have received the booster dose. Similarly, 95.0% of individuals ages 75 + have received at least one dose, 85.5% have been fully vaccinated and 68.9% have received the booster dose.

Consistent with previous studies’ findings [13], we also found that educational attainment was a significant positive predictor for COVID-19 vaccine uptake. Individuals with higher educational attainment may have better access to accurate vaccine information and the health literacy to understand that health information and navigate initially complex systems for obtaining the vaccine. In a survey done by the Census in December 2021, 49.6% of individuals were concerned with the vaccine side effects, 42.4% of individuals did not trust the vaccine and 35.4% of individuals did not trust the government [33]. Individuals with higher educational attainment may also be able to better understand how the vaccine works, which can reduce fear surrounding possible side effects of the vaccine. According to a Census report, the unvaccinated adults who were most hard to reach were more likely to be young adults under the age of 50, non-white, and unmarried [32]. These adults presented lower levels of education and economic stability, tending to manifest as increased difficulty meeting daily expenses. They were also more likely to report disabilities such as difficulty seeing, hearing, transporting, remembering, or having complete impairment, which made it harder for them to access the vaccine.

In our study, occupation was also a major predictor for COVID-19 vaccine uptake. Those who worked in the healthcare industry as a practitioner or supporter were significantly more likely to have received one or more doses of the COVID-19 vaccine than those who worked in other professional roles. Healthcare workers were on the front lines during the initial stages of the pandemic, and were in the highest priority group to receive the vaccine when it was first being distributed. The daily exposure to high-risk individuals and severe COVID-19 presentations may have pushed those working in healthcare to take the vaccine readily when it was offered to them [34]. Many hospitals and healthcare facilities also instituted a vaccine mandate for all employees, which may explain the significant association between those working in healthcare and vaccine uptake. As of September 21, 2021, at least 174 health systems required all of their employees to be vaccinated against COVID-19 [35], and a recent ruling by the Supreme Court enabled the Centers for Medicare and Medicaid Services to require all Medicare and Medicaid service providers to be vaccinated [36]. This may explain how the significant association between vaccination and working in healthcare is consistent in both from January 2021 to May 2021 and June 2021 to February 2022. Those who worked in arts, entertainment, and media also demonstrated a significant increase in vaccine uptake. As the pandemic waned and media production went back to in-person work, many large entertainment companies required that their employees be vaccinated and routinely tested to ensure worker safety, including The Walt Disney Company and NBC Universal [37]. This may have contributed to the associations observed between vaccine uptake and working in arts, entertainment, and media industries.

Those who worked in primarily blue-collar industries such as construction, installation & repair, and transportation & material moving demonstrated low rates of vaccine uptake and high rates of vaccine hesitancy across both time periods observed. A study by Carnegie Mellon University in collaboration with the University of Pittsburgh found that high-hesitancy occupations such as construction reported a lack of trust in both the COVID-19 vaccine and the U.S. government as a key driver for hesitancy. These individuals also expressed the belief that they do not need the vaccine, which may be due to the fact that much work in construction, repair, and transportation occurs either primarily outdoors or in uncrowded settings [38]. 

Moreover, our study found differential rates of vaccination and vaccine hesitancy across racial/ethnic groups. Whites and Asians were more likely to be vaccinated and have lower rates of vaccine hesitancy in comparison with blacks, multiracial individuals as well as Native American/Alaska Native, and Native Hawaiian/other Pacific Islanders. These differences could be due to differences in vaccination access and distribution. Initially neighborhoods with greater shares of whites and Asians had higher vaccination rates [39, 40]. Our results are in alignment with CDC published vaccination rates that report Asians having the highest rate of fully vaccinated (59.8%) and proportion of booster doses (65.4%), and Blacks having the lowest rate of fully vaccinated (40.6%), and the Hispanic/Latino population having the lowest proportion of booster doses (38.5%) [9].

Also, we found that individuals who identify as “other gender” had lower vaccination rates and higher rates of vaccine hesitancy than females and males. Individuals who identify with genders different than female and male had historically encountered challenges when accessing, trusting, and obtaining health care services. Thus, not trusting the COVID-19 vaccine and the system could be the reason why they presented lower rates of vaccination and higher rates of vaccine hesitancy. However, other studies have not found differences in vaccination or vaccine confidence by gender identity [39]. Exploring vaccination patterns among gender minorities is highly understudied and warrants further investigation.

As one of the most efficient ways of protecting ourselves and others from COVID-19, wearing masks was set as an outcome in our study. At the start of the COVID pandemic, besides isolation, mask-wearing was the widely recommended method of combating infection. However, the masking recommendation that was being encouraged by much of the scientific community was very quickly met with anti-masking resistance. Similar resistance was also expressed at the onset of the vaccine development. Our study highlighted an inverse correlation between mask-wearing and vaccine hesitancy, which has also been identified by other studies [25, 41]. This association might indicate that the faction of the community that is less likely to wear masks and more likely to express vaccine hesitancy also has have skepticism about the severity of the pandemic or mistrust of the government and the scientific community [42].

We found that older people were more likely to wear masks most or all the time in public compared to young adults. The higher chance of facing more severe health issues from COVID-19 could be one reason why older people valued mask wearing more than other age groups [43]. We also found that compared to male respondents, female respondents were more likely to wear masks most of the time or all the time. A previous study found that males were more likely to perceive face masks as infringing on their rights and independence compared to females [44]. Racial/ethnic minorities were more likely to wear masks most of the time or all the time compared whites, which aligns with previous literature [45]. Possible reason could be awareness of higher COVID-19 risk and severity of illness among racial/ethnic minorities [46]. According to data from the CDC, Black and Latina/o adults are more likely to become infected, be hospitalized due to severe illness, and die from COVID-19 complications when compared with White people. Asian Americans are also more likely to be become infected and be hospitalized when compared with White people. Lower mask wearing was also seen in protective service, construction, and installation and repair occupations. These groups also had lower vaccination rates and higher vaccine hesitancy. For many of them who worked outdoors, they might perceive lower transmission risk [47]. Additionally, political polarization around mask wearing [48] could further help explain patterns seen across occupations.

With the exception of bachelor’s degrees which were associated with higher vaccination rate and lower vaccine hesitancy, our study did not identify contextual factors at the ZIP code level that significantly predict COVID vaccination status or vaccine hesitancy. For mask wearing, percent Hispanic and sidewalks were associated with higher odds of wearing masks most or all the time. This is consistent with the finding that racial/ethnic minorities have higher mask wearing [45]. The presence of sidewalks may indicate greater opportunities for social interactions and hence contagion, which may lead individuals to wear masks. However, unexpectedly, civilian employment rate and mixed land use was associated with lower odds of mask wearing because a previous analysis linked these with more COVID cases [28]. Neighborhood data offers a granular geographical context and is known as a significant determinant of health outcomes. Future studies should expand upon our research by further investigating neighborhood level determinants that could influence behaviors around COVID-19 vaccines and mask-wearing.

### Study strengths and limitations

Our study analyzed millions of responses to the U.S. COVID-19 Trends and Impact Survey which provided valuable insights into the behaviors and attitudes of the U.S. population during the pandemic. Additional strengths included examining responses to this daily survey for over a year’s time (January 2021 to February 2022) which permitted time stratified analyses to examine differential trends across time for related COVID-19 behaviors and attitudes. Included in models were both individual and ZIP code level predictors.

Nonetheless, our study was subject to study limitations. For instance, political affiliations were not assessed in the survey but could have helped explain certain patterns seen for COVID-19 vaccination and masking behaviors across certain demographics and occupations [49–51]. Moreover, throughout the survey, respondents are able to refuse to answer, say “don’t know” or select “other” category. Thus, as with other survey research, our analysis was limited to what respondents chose to report or were comfortable reporting via an online platform. Social desirability is a concern, especially when survey participants are asked to report on something that is sensitive or activates considerations of social acceptability or norms. Respondents may feel varying pressures to report certain beliefs or behaviors (e.g., mask wearing, vaccine hesitancy) depending on how they think others will judge them and these reports may or may not reflect their own beliefs or behaviors. Moreover, while we were able to examine individual and some contextual factors related to COVID-19 behaviors and attitudes, our list was not exhaustive. For example, weather conditions were not included in the analysis and could have impacted people’s willingness to get vaccinated. Across the country, mass vaccinations occurring in outdoor stadiums, churches and other public locations could have been hindered by adverse weather events and the likelihood of these weather events can vary geographically. Future studies further investigating geographical differences may want to include weather conditions.

Furthermore, although we utilized over a year of data, given that respondents are not followed through time, our analysis was cross-sectional and hence causal inference is limited. Because of the cross-sectional nature of the survey and analysis, we were unable to examine changes in vaccination status (e.g., individuals continuing to get their vaccine series or booster shots). Moreover, the survey did not ask participants to report on the particular vaccine product (e.g., Johnson & Johnson, Pfizer, Moderna) and hence analyses could not be stratified to examine whether predictors differentially uptake of particular COVID-19 vaccines. While we stratified analyses by time period, we did not account for time varying characteristics including COVID-19 variants which differed in regards to transmissibility, clinical disease presentation,

as well as effectiveness of public health and social measures or available diagnostics, vaccines, and therapeutics. Future investigations into how COVID-19 variants influenced vaccination status, vaccine hesitancy, and mask wearing is warranted.

## Conclusion

Findings have demonstrated that there are a variety of factors that influence individuals’ COVID-19 vaccine uptake, vaccine hesitancy, and mask wearing. Our study expands upon the emerging research [24, 25] finding ties between mask wearing and vaccine acceptance. Major predictors for COVID-19 prevention behaviors include age, education, occupation, race/ethnicity, language, and community socioeconomic status. Associations found in earlier phases of the pandemic were generally found to also be present later in the pandemic, indicating stability in inequities. Inequities in these important outcomes suggest more work is needed to bridge gaps to ensure that the burden of COVID-19 risk does not disproportionately fall upon certain subgroups.

## Data Availability

The Delphi Group at Carnegie Mellon University U.S. COVID-19 Trends and Impact Survey, in partnership with Facebook is available publicly, https://dataforgood.facebook.com/dfg/tools/covid-19-trends-and-impact-survey. The race/ethnicity variable generated and analyzed during the current study are not publicly available but can be requested through a restricted data use agreement (https://cmu-delphi.github.io/delphi-epidata/symptom-survey/data-access.html). ZIP code level sociodemographic data are publically available from the American Community Survey.

## References

[1] CDC Museum COVID-19 Timeline. [https://www.cdc.gov/museum/timeline/covid19.html#:~:text=January%2020%2 C%202020%20CDC,18%20in%20Washington%20state.].

[2] World Health Organization. WHO Coronavirus (COVID-19) Dashboard. 2022.

[3] Gostin LO, Cohen IG, Koplan JP (2020). Universal Masking in the United States: The Role of Mandates, Health Education, and the CDC. JAMA.

[4] States. cities and counties have largely eased or ended mandates https://www.aarp.org/health/healthy-living/info-2020/states-mask-mandates-coronavirus.html.

[5] Approves First FDA. COVID-19 Vaccine https://www.fda.gov/news-events/press-announcements/fda-approves-first-covid-19-vaccine.

[6] COVID-19 Vaccines. https://www.hhs.gov/coronavirus/covid-19-vaccines/index.html.

[7] Possible Side Effects. After Getting a COVID-19 Vaccine https://www.cdc.gov/coronavirus/2019-ncov/vaccines/expect/after.html.

[8] Do COVID-19. vaccines protect against the variants? https://www.mayoclinic.org/coronavirus-covid-19/covid-variant-vaccine.

[9] COVID Data Tracker Weekly Review. https://www.cdc.gov/coronavirus/2019-ncov/covid-data/covidview/index.html.

[10] Troiano G, Nardi A (2021). Vaccine hesitancy in the era of COVID-19. Public Health.

[11] Dror AA, Eisenbach N, Taiber S, Morozov NG, Mizrachi M, Zigron A, Srouji S, Sela E (2020). Vaccine hesitancy: the next challenge in the fight against COVID-19. Eur J Epidemiol.

[12] Larson HJ, Jarrett C, Eckersberger E, Smith DM, Paterson P (2014). Understanding vaccine hesitancy around vaccines and vaccination from a global perspective: a systematic review of published literature, 2007–2012. Vaccine.

[13] Khubchandani J, Sharma S, Price JH, Wiblishauser MJ, Sharma M, Webb FJ (2021). COVID-19 vaccination hesitancy in the United States: a rapid national assessment. J Community Health.

[14] Center SHADA. New Evidence from the Household Pulse Survey: Vaccine Hesitancy Decreased During the First Three Months of 2021. In.; 2022.

[15] Leach R. Vaccine Hesitancy and Compassion Fatigue: Infection Preventionists Battle Both at Once. Infection Control Today 2021, 25(7).

[16] Allen JD, Feng W, Corlin L, Porteny T, Acevedo A, Schildkraut D, King E, Ladin K, Fu Q, Stopka TJ (2021). Why are some people reluctant to be vaccinated for COVID-19? A cross-sectional survey among U.S. Adults in May-June 2020. Prev Med Rep.

[17] Hamel E, Lopes L, Sparks G, Kirzinger A, Kearney A, Stokes M. Brodie aM: KFF COVID-19 Vaccine Monitor: September 2021. In. kff.org; 2022.

[18] Park HK, Ham JH, Jang DH, Lee JY, Jang WM. Political Ideologies, Government Trust, and COVID-19 Vaccine Hesitancy in South Korea: A Cross-Sectional Survey. Int J Environ Res Public Health 2021, 18(20). 10.3390/ijerph182010655 PMC8536119 34682401

[19] Pink SL, Chu J, Druckman JN, Rand DG, Willer R: Elite party cues increase vaccination intentions among Republicans. *Proceedings of the National Academy of Sciences* 2021, 118(32):e2106559118. 10.1073/pnas.2106559118 PMC8364165 34312254

[20] Dror AA, Daoud A, Morozov NG, Layous E, Eisenbach N, Mizrachi M, Rayan D, Bader A, Francis S, Kaykov E (2021). Vaccine hesitancy due to vaccine country of origin, vaccine technology, and certification. Eur J Epidemiol.

[21] State COVID-19 Data and Policy Actions [https://www.kff.org/report-section/state-covid-19-data-and-policy-actions-policy-actions/].

[22] State Efforts to Ban or Enforce COVID-19 Vaccine Mandates. and Passports https://www.nashp.org/state-lawmakers-submit-bills-to-ban-employer-vaccine-mandates/.

[23] Brüssow H, Zuber S (2022). Can a combination of vaccination and face mask wearing contain the COVID-19 pandemic?. Microb Biotechnol.

[24] Latkin CA, Dayton L, Yi G, Colon B, Kong X (2021). Mask usage, social distancing, racial, and gender correlates of COVID-19 vaccine intentions among adults in the US. PLoS ONE.

[25] Bukhari A, Adeyinka DA, McCutcheon J, Kallio N, Muhajarine N (2022). Characteristics Associated with the Dual Behavior of Mask Wearing and Vaccine Acceptance: A Pooled Cross-Sectional Study among Adults in Saskatchewan. Int J Environ Res Public Health.

[26] Salomon JA, Reinhart A, Bilinski A, Chua EJ, Motte-Kerr WL, Rönn MM, Reitsma M, Morris KA, LaRocca S, Farag T, Kreuter F, Rosenfeld R, Tibshirani RJ. The US COVID-19 Trends and Impact Survey: Continuous real-time measurement of COVID-19 symptoms, risks, protective behaviors, testing, and vaccination. Proceedings of the National Academy of Sciences. 2021; 118 (51) e2111454118. 10.1073/pnas.2111454118 10.1073/pnas.2111454118 PMC8713763 34903656

[27] Methodology Report for the COVID-19 Trends and. Survey I. https://dataforgood.facebook.com/dfg/resources/CTIS-methodology-report.

[28] Nguyen QC, Huang Y, Kumar A, Duan H, Keralis JM, Dwivedi P, Meng H-W, Brunisholz KD, Jay J, Javanmardi M (2020). Using 164 Million Google Street View Images to Derive Built Environment Predictors of COVID-19 Cases. Int J Environ Res Public Health.

[29] Nguyen QC, Belnap T, Dwivedi P, Deligani AHN, Kumar A, Li D, Whitaker R, Keralis J, Mane H, Yue X (2022). Google Street View Images as Predictors of Patient Health Outcomes, 2017–2019. Big Data and Cognitive Computing.

[30] Nguyen TT, Nguyen QC, Rubinsky AD, Tasdizen T, Deligani AHN, Dwivedi P, Whitaker R, Fields JD, DeRouen MC, Mane H (2021). Google Street View-Derived Neighborhood Characteristics in California Associated with Coronary Heart Disease, Hypertension, Diabetes. Int J Environ Res Public Health.

[31] Savoia E, Piltch-Loeb R, Goldberg B, Miller-Idriss C, Hughes B, Montrond A, Kayyem J, Testa MA. Predictors of COVID-19 Vaccine Hesitancy: Socio-Demographics, Co-Morbidity, and Past Experience of Racial Discrimination. Vaccines 2021, 9(7). 10.3390/vaccines9070767 PMC8310049 34358184

[32] COVID-19 Vaccinations in the United States. https://covid.cdc.gov/covid-data-tracker/#vaccinations_vacc-total-admin-rate-total.

[33] Household Pulse Survey Shows Many Don’t Trust COVID Vaccine. Worry About Side Effects [https://www.census.gov/library/stories/2021/12/who-are-the-adults-not-vaccinated-against-covid.html].

[34] Li M, Luo Y, Watson R, Zheng Y, Ren J, Tang J, Chen Y. Healthcare workers’ (HCWs) attitudes and related factors towards COVID-19 vaccination: a rapid systematic review. Postgraduate Medical Journal 2021:postgradmedj-2021-140195. 10.1136/postgradmedj-2021-140195 37319159

[35] As. CMS’ requirement looms, at least 174 health systems currently mandate vaccination for their workforces.

[36] COVID-19 Vaccination Requirements for Health Care Providers and. Suppliers. https://www.cms.gov/files/document/covid-19-health-care-staff-vaccination-requirements-infographic.pdf.

[37] From Amex to Walmart. here are the companies mandating the Covid vaccines for employees [https://www.nbcnews.com/business/business-news/amex-walmart-are-companies-mandating-covid-vaccine-employees-rcna11049].

[38] King WC, Rubinstein M, Reinhart A, Mejia R (2021). COVID-19 vaccine hesitancy January-May 2021 among 18–64 year old US adults by employment and occupation. Prev Med Rep.

[39] María Nápoles A, Stewart AL, Strassle PD, Quintero S, Bonilla J, Alhomsi A, Santana-Ufret V, Maldonado AI (2021). Pérez-Stable EJ: Racial/ethnic disparities in intent to obtain a COVID-19 vaccine: A nationally representative United States survey. Prev Med Rep.

[40] Sacarny A, Daw JR (2021). Inequities in COVID-19 Vaccination Rates in the 9 Largest US Cities. JAMA Health Forum.

[41] Latkin CA, Dayton L, Yi G, Konstantopoulos A, Boodram B: Trust in a COVID-19 vaccine in the US: A social-ecological perspective. *Social science & medicine (*1982*)* 2021, 270:113684. 10.1016/j.socscimed.2021.113684 PMC7834519 33485008

[42] Both Republicans. and Democrats cite masks as a negative effect of COVID-19, but for very different reasons [https://www.pewresearch.org/fact-tank/2020/10/29/both-republicans-and-democrats-cite-masks-as-a-negative-effect-of-covid-19-but-for-very-different-reasons/].

[43] Haischer MH, Beilfuss R, Hart MR, Opielinski L, Wrucke D, Zirgaitis G, Uhrich TD, Hunter SK (2020). Who is wearing a mask? Gender-, age-, and location-related differences during the COVID-19 pandemic. PLoS ONE.

[44] Howard MC (2021). Gender, face mask perceptions, and face mask wearing: Are men being dangerous during the COVID-19 pandemic?. Pers Indiv Differ.

[45] Hearne BN, Niño MD (2022). Understanding how race, ethnicity, and gender shape mask-wearing adherence during the COVID-19 pandemic: evidence from the COVID impact survey. J racial ethnic health disparities.

[46] Raifman MA, Raifman JR (2020). Disparities in the population at risk of severe illness from COVID-19 by race/ethnicity and income. Am J Prev Med.

[47] Freidin E, Acera Martini L, Senci CM, Duarte C, Carballo F (2022). Field observations and survey evidence to assess predictors of mask wearing across different outdoor activities in an Argentine city during the COVID-19 pandemic. Appl Psychology: Health Well‐Being.

[48] Lang J, Erickson WW, Jing-Schmidt Z (2021). # MaskOn!# MaskOff! Digital polarization of mask-wearing in the United States during COVID-19. PLoS ONE.

[49] Wood D, Brumfiel G. Pro-Trump counties continue to suffer far higher COVID death tolls. In: NPR. 2022.

[50] Milligan MA, Hoyt DL, Gold AK, Hiserodt M, Otto MW (2022). COVID-19 vaccine acceptance: influential roles of political party and religiosity. Psychol health Med.

[51] Young DG, Rasheed H, Bleakley A, Langbaum JB (2022). The politics of mask-wearing: Political preferences, reactance, and conflict aversion during COVID. Soc Sci Med.

